# Frequency and Impact of Cardiology Evaluation Following Perioperative Myocardial Infarction

**DOI:** 10.1002/clc.70120

**Published:** 2025-03-21

**Authors:** Anthony Hung, R. Parker Ward, Daniel S. Rubin

**Affiliations:** ^1^ Pritzker School of Medicine University of Chicago Chicago Illinois USA; ^2^ Department of Medicine University of Chicago Chicago Illinois USA; ^3^ Department of Anesthesia and Critical Care University of Chicago Chicago Illinois USA

**Keywords:** cardiology, consultation, echocardiography, myocardial infarction, perioperative

## Abstract

**Background:**

Perioperative myocardial infarction (PMI) after noncardiac surgery results in significant morbidity and mortality. While comprehensive management, including imaging and guideline‐directed medical therapy (GDMT), improves outcomes, utilization of these strategies and the impact of physician evaluation on their utilization are unknown. This study evaluates the frequency of cardiology evaluation after PMI and its association with guideline‐recommended care.

**Methods:**

Using IBM MarketScan (2016–2021), we analyzed claims for patients ≥ 45 years old with PMI during or after major noncardiac surgery. We examined the relationship between cardiology evaluation and post‐PMI care using three regression models: (1) a Poisson model for GDMT class prescriptions filled within 3 months post‐discharge, and logistic models for (2) ischemic testing and (3) echocardiography during hospitalization or within 3 months post‐discharge.

**Results:**

Among 5660 patients with PMI (mean age 68, 56.9% male, 27.2% with STEMI), 19% were not evaluated by a cardiologist. Patients with cardiology evaluation were more likely to receive at least one GDMT prescription after PMI (78.8% vs 74.0%, *p* < 0.001). Cardiology evaluation was also associated with an increased likelihood of ischemic testing (38.2% vs 23.0%, *p* < 0.001) and echocardiography (75.9% vs 53.6%, *p* < 0.001).

**Conclusion:**

One in five PMI patients lacks cardiology evaluation, and evaluation is associated with an increased likelihood of recommended management after PMI. Future studies should explore whether cardiology evaluation and management strategies impact patient outcomes.

## Graphical abstract

We analyzed the frequency of cardiology evaluation after perioperative myocardial infarction and its association with management in 5660 patients. One in five patients lacked cardiology evaluation. Cardiology evaluation was associated with an increased likelihood of guideline‐directed medical therapy (78.8% vs 74.0%), ischemic testing (38.2% vs 23.0%), and echocardiography (75.9% vs 53.6%).

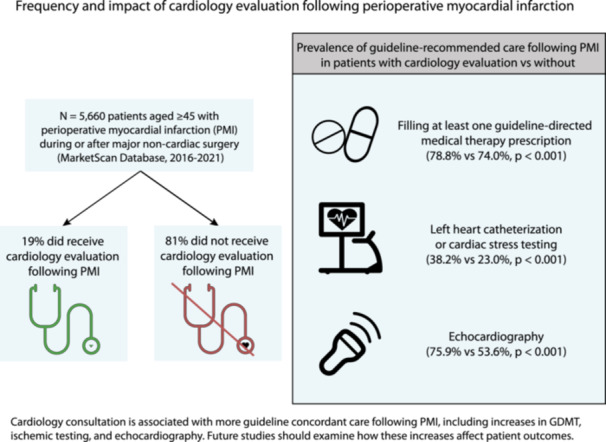

## 1 Introduction

Perioperative myocardial infarction (PMI) is a significant and prevalent complication following noncardiac surgery, with substantial implications for adverse patient outcomes. Studies indicate that PMI occurs in 0.76% to 5% of patients undergoing major noncardiac surgery [1, 2, 3]. The mortality rate within 1 year post‐surgery for patients experiencing PMI ranges from 10% to 37% [4, 5, 6, 7]. Those who survive this acute event remain at an elevated risk for recurrent MI, heart failure, and mortality [6].

Guideline‐directed management of MI patients is critical to improve outcomes. Current guidelines recommend several strategies, including echocardiography, ischemic testing, and guideline‐directed medical therapy (GDMT) [8, 9]. GDMT typically includes antiplatelet agents (aspirin, P2Y12 receptor inhibitors), statins, ACE inhibitors or angiotensin receptor blockers, and beta‐blockers. GDMT has been shown to significantly improve patient outcomes, including reducing mortality, following MI [10, 11, 12].

Despite the established guidelines, there is limited information regarding the real‐world implementation of these management strategies following PMI. Moreover, the frequency of cardiology evaluation in real‐world practice, and the impact cardiology evaluation has on the implementation of these guideline‐based management strategies after PMI is unknown. The gap between guideline recommendations and clinical practice, often referred to as the “implementation gap,” is a well‐recognized phenomenon in cardiovascular care [13, 14, 15]. Identifying and addressing the barriers to guideline adherence in the context of PMI may significantly improve patient care and outcomes.

To address these knowledge gaps, we conducted a study using a U.S. insurance claims database to characterize the management of PMI from 2016 to 2021. This study has two aims: (1) to assess the frequency and patterns of GDMT, cardiac workup strategies, and cardiology evaluation following PMI, and (2) to identify whether cardiology evaluation is associated with the frequency of GDMT and cardiac workup.

## 2 Methods

### 2.1 Data Source

Data for these analyses came from the Truven Health MarketScan Databases (2015–2021) [16], which provide deidentified, longitudinal data on medical services and prescription drug claims. These databases include individuals with employer‐sponsored insurance, their dependents, and Medicare‐eligible retirees with supplemental insurance. MarketScan tracks claims across sites and over time, covering approximately 6 million U.S. individuals, with a nationally representative sex distribution (49% male).

### 2.2 Study Population

**Figure 1:**
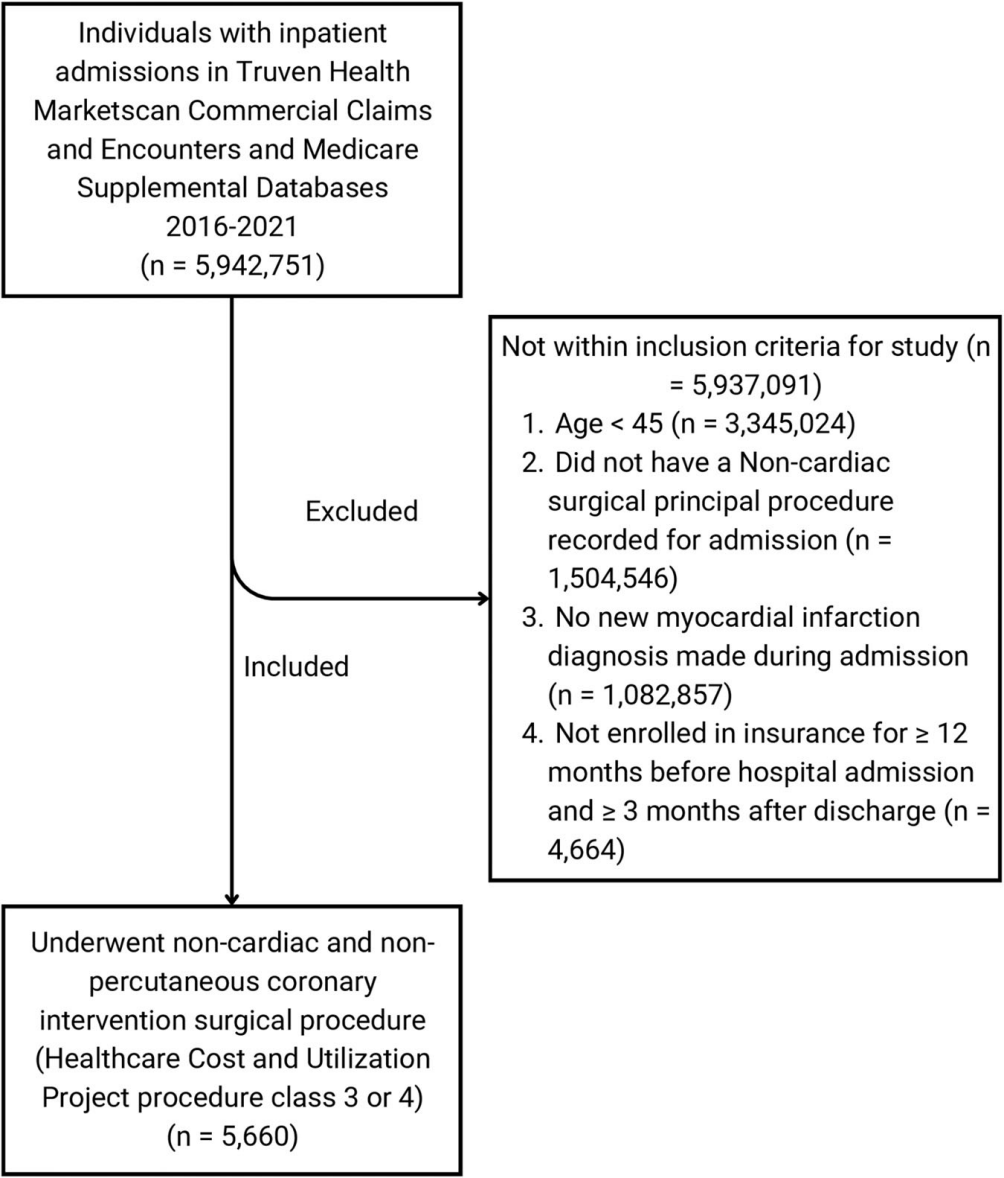
Study flow diagram showing inclusion and exclusion criteria.

Patients aged 45 and older in the Health MarketScan Commercial Claims and Encounters and Medicare Supplemental Databases were included in the study cohort if they (1) had an inpatient admission falling between January 1, 2016 and September 30, 2021, (2) had a major noncardiac operating room procedure as their principal procedure [17, 18], and (3) received a diagnosis of a new myocardial infarction during their admission (Figure 1).

Specifically for criteria (2), admissions with principal procedure codes in Healthcare Cost and Utilization Project procedure classes 3 or 4, corresponding to major diagnostic and major therapeutic procedures, respectively, that did not correspond to procedures involving the heart, great vessels, or coronary vessels, were included (Table S1). For criteria (3), myocardial infarction was defined as a diagnosis corresponding to ICD‐9 code 410 or ICD‐10 code I21. There were no individuals with missing data.

### 2.3 Outcomes

The primary outcome was the number of GDMT medication classes with prescriptions filled within the 3 months post‐discharge from a hospitalization with a PMI. This time frame was chosen to assess short‐term guideline adherence while allowing for potential delays in follow‐up care and medication initiation or prescription filling. Four medication classes were defined as GDMT classes: platelet inhibitors (consisting of P2Y12 receptor inhibitors or aspirin), statins, ACE inhibitors or angiotensin receptor blockers, and beta blockers, and prescriptions were determined using National Drug Code numbers (Table S2). As MarketScan data only includes records on medications for which prescriptions were written and filled in the outpatient setting, the purchase of aspirin by a patient over‐the‐counter without the use of a prescription would not be captured by MarketScan. Two secondary outcomes were also assessed: (1) stress testing or left heart catheterization within the index admission to 3 months post‐discharge and (2) echocardiography within the same period, both identified using CPT codes (Table S3).

### 2.4 Patient Characteristics and Covariates

Patient age, sex, and length of stay of the index admission containing a PMI were obtained for each patient. The clinical domain of the principal procedure for the index admission with a PMI was determined for each patient using the Healthcare Cost and Utilization Project Procedure Classes Refined for ICD‐10‐PCS, v2023.1 (Table S1). To ensure statistical stability in our analyses, procedural categories with fewer than 50 patients (Ear, Nose, and Throat Procedures *n* = 30, Endocrine Procedures *n* = 23, Eye Procedures *n* = 9, Peripheral Nervous System Procedures *n* = 19, Pregnancy‐Related Procedures *n* = 6, and Skin and Breast Procedures *n* = 38) were combined into an ‘Other Procedures’ category. This approach avoids unreliable coefficient estimates from categories with very small sample sizes while maintaining clinical relevance in our analyses. Emergent procedures were defined as procedures occurring during an admission initiated with a service subcategory code that corresponded to an Emergency Room visit. The presence of evaluation by a provider falling into a specialty of “cardiology,” “family medicine,” or “internal medicine, not elsewhere classified” was determined separately for each patient during their index admission and for the 3‐month period following discharge. The presence of diagnoses of cardiac complications (supraventricular tachycardia, cardiogenic shock, atrial fibrillation, or acute heart failure) and noncardiac complications (severe sepsis, septic shock, hypovolemic shock, respiratory failure, or pulmonary embolism) made during the index admission were identified using ICD diagnosis codes (Table S4). Preoperative and perioperative risk factors recorded within the year before index admission, including ischemic heart disease, heart failure, chronic kidney disease (CKD) stage 3 or higher, and prior stroke or transient ischemic attack were identified using ICD diagnosis codes (Table S4). The presence of insulin‐dependent diabetes was identified using the presence of a filled outpatient prescription for insulin within the year before index admission. Modified Revised Cardiac Risk Index scores [19] were calculated using the above preoperative risk factors, with CKD 3 or higher as a proxy for creatinine > 2.0 and with high‐risk surgery defined as intraperitoneal, intrathoracic, or supra‐inguinal vascular surgery. The Clinical Classifications Software Refined categories were used to identify the high‐risk surgical procedures (Table S5) [20]. Using ICD codes (Table S4), we classified PMI events based on the diagnostic codes assigned to patients during hospitalization, which included STEMI, NSTEMI, and type 2 myocardial infarction. As these diagnoses are not mutually exclusive, patients may have received multiple MI‐related codes during their admission.

### 2.5 Statistics

All statistical analyses were completed using R statistical software environment version 4.2.2 [21]. Baseline characteristics were reported as mean and standard deviation (continuous variables) or as proportions (categorical variables). Continuous variables were compared using the Student's *t*‐tests or ANOVA tests, and categorical variables were compared with chi‐square tests. Due to the non‐normal distribution of length of stay data, we reported this variable as median with interquartile range and used non‐parametric Mann‐Whitney *U* tests for two‐group comparisons and Kruskal‐Wallis tests for multi‐group comparisons. To address the potential for Type I error due to multiple comparisons, we applied the Benjamini‐Hochberg procedure to control the False Discovery Rate (FDR) at 0.05 for all statistical tests. Statistical significance was considered with an adjusted *p*‐value less than 0.05.

### 2.6 Model of Frequency of Guideline Directed Medical Therapy Following Perioperative Myocardial Infarction

We fit a Poisson model with the glm function in R to assess the association between evaluation by a cardiologist physician during or after hospitalization with PMI and the number of GDMT drug categories (platelet inhibitors, ACE inhibitors/ARBs, beta‐blockers, or statins) with filled prescriptions within 3 months post‐discharge after PMI, while accounting for demographics and patient history. We selected Poisson regression because the number of drug categories is a count variable. To verify the appropriateness of this modeling approach, we conducted a Pearson chi‐square test for overdispersion, which showed no evidence of overdispersion (χ² = 5412.21, df = 5659, *p* = 0.991). The mean number of medication classes was 1.88 with a variance of 1.80, yielding a dispersion ratio of 0.96, which is close to 1.0 and confirms that the Poisson distribution appropriately fits our data.

Covariates included age, sex, length of hospital stay, emergency status of admission, clinical domain of principal procedure, and presence of filled prescriptions for ACE inhibitors/ARBs, beta‐blockers, aspirin, statins, and P2Y12 receptor inhibitors in the year before the index admission. Wald tests were used to assess the significance of each covariate, testing whether coefficients differed from zero, and *p*‐values were adjusted to an FDR of 0.05 using the Benjamini‐Hochberg procedure.

To assess the clinical significance of differences in GDMT utilization, we analyzed the association between cardiology evaluation and receipt of two or more GDMT medication classes, which represents a clinically meaningful threshold of guideline adherence. We calculated the proportion of patients receiving at least two GDMT medication classes stratified by cardiology evaluation status. The absolute risk difference between groups was determined, along with its 95% confidence interval calculated using the standard error formula for the difference between two proportions. The statistical significance of the difference in proportions was assessed using a chi‐square test.

### 2.7 Models of the Frequency of Ischemic Testing and Echocardiography Following Perioperative Myocardial Infarction

We created two separate logistic regression models with the glm function in R to evaluate the association between various factors and the likelihood of undergoing specific management strategies in the period spanning the index admission to 3 months following discharge from an admission with a post‐PMI: left heart catheterization or stress testing, and echocardiography. For each model, we included clinical and demographic variables including clinical domain of primary procedure, age, sex, comorbidities (ischemic heart disease, heart failure, chronic kidney disease stage 3 or higher, cerebrovascular disease, and insulin‐dependent diabetes), length of stay, emergency status, cardiac complications, noncardiac complications, evaluation by cardiology, family medicine, and internal medicine physicians, prior use of catheterization, stress tests, and echocardiography in the year before the index admission. The binary outcome variables for the two models were (1) Undergoing catheterization or stress testing during or 3 months after the inpatient stay and (2) Undergoing echocardiography during or 3 months after the inpatient stay. For both models, p‐values for each covariate were derived from Wald tests, and *p*‐values were adjusted to an FDR of 0.05 using the Benjamini‐Hochberg procedure.

## 3 Results

### 3.1 Study Population

A total of 1,093,181 patients underwent *a major* Noncardiac operating room procedure between January 1, 2016 and September 30, 2021, with 10,324 patients (0.94%) experiencing a PMI. Patients with insufficient insurance coverage (*n* = 4664) were excluded from the final analysis. The study cohort consisted of reports from hospitalizations for 5660 patients, and characteristics of these patients are presented in Table 1. The mean age was 68 years (SD 12.2 years), and 56.9% (*n* = 3223) were male. Of PMIs, 27.2% were STEMIs, 58.2% were NSTEMIs, and 19.7% were Type 2 MIs. Only 18.6% (*n* = 1053) of the admissions were nonelective surgical procedures.

**Table 1:** Baseline characteristics among patients with perioperative AMI after major noncardiac surgery.

Characteristics	Mean/Median/Count
Age	68 (12.2)
Length of stay (days)	8 [4–14]
Sex = Male	3223 [56.9%]
Year	
2016	1323 [23.4%]
2017	1009 [17.8%]
2018	746 [13.2%]
2019	812 [14.3%]
2020	992 [17.5%]
2021	778 [13.7%]
Urgency of Procedure = Emergent	1053 [18.6%]
Type of surgery	
Vascular Procedures	1472 [26%]
Central Nervous System Procedures	154 [2.72%]
Female Reproductive System Procedures	69 [1.22%]
Gastrointestinal System Procedures	751 [13.3%]
General Region Procedures	138 [2.44%]
Hepatobiliary and Pancreas Procedures	342 [6.04%]
Lymphatic and Hemic System Procedures	67 [1.18%]
Male Reproductive System Procedures	60 [1.06%]
Musculoskeletal, Subcutaneous Tissue, and Fascia Procedures	1907 [33.7%]
Respiratory System Procedures	379 [6.7%]
Urinary System Procedures	196 [3.46%]
Other Procedures	125 [2.21%]
Complications occurring during hospitalization with PMI	
Cardiac complication	2037 [36%]
Supraventricular tachycardia	211 [3.73%]
Cardiogenic shock	180 [3.18%]
Atrial fibrillation	1299 [23%]
Acute heart failure	837 [14.8%]
Noncardiac complication	1952 [34.5%]
Severe sepsis	224 [3.96%]
Septic shock	305 [5.39%]
Hypovolemic shock	68 [1.2%]
Respiratory failure	1680 [29.7%]
Pulmonary embolus	231 [4.08%]
Evaluation by physician during admission with PMI or in 3 months post discharge	
Cardiologist	4582 [81%]
Family Medicine	3267 [57.7%]
Internal Medicine (not elsewhere classified)	4254 [75.2%]
GDMT class prescription filled in 12 months before admission	
Aspirin	261 [4.61%]
P2Y12 receptor inhibitor	1283 [22.7%]
ACE inhibitor/ARB	3009 [53.2%]
Beta Blocker	2954 [52.2%]
Statin	3141 [55.5%]
GDMT class prescription filled in 3 months after discharge	
Aspirin	461 [8.14%]
P2Y12 receptor inhibitor	1484 [26.2%]
ACE inhibitor/ARB	2373 [41.9%]
Beta Blocker	3389 [59.9%]
Statin	3156 [55.8%]
Prior cardiac evaluation	
Cardiac stress testing in year before admission with PMI	1012 [17.9%]
Cardiac stress testing during admission with PMI or in three months after discharge	698 [12.3%]
Left heart catheterization in year before admission with PMI	705 [12.5%]
Left heart catheterization during admission with PMI or in three months after discharge	1501 [26.5%]
Echocardiography in year before admission with PMI	2305 [40.7%]
Echocardiography during admission with PMI or in three months after discharge	4057 [71.7%]
Prior Comorbidities	
Ischemic heart disease	2628 [46.4%]
Heart failure	1366 [24.1%]
Insulin‐dependent diabetes mellitus	993 [17.5%]
Stroke or transient ischemic attack	455 [8.04%]
Chronic kidney disease stage 3 or higher	1095 [19.3%]
RCRI score	1 [0–2]

*Note:* Mean (standard deviation) is reported for each numeric variable, and count [percent of total] is reported for each categorical variable. Median [IQR] was reported for length of stay and RCRI score due to the non‐normality of these numeric variables.

Abbreviations: GDMT, guideline‐directed medical therapy; PMI, perioperative myocardial infarction; RCRI, revised cardiac risk index.

Before the index admission, 46.4% (*n* = 2628) of patients had a history of ischemic heart disease, 24.1% (*n* = 1366) had heart failure, 8.04% (*n* = 455) had a prior stroke or transient ischemic attack, 19.3% (*n* = 1095) had chronic kidney disease stage 3 or higher, and 17.5% (*n* = 993) had insulin‐dependent diabetes. Patients in the general cohort had a median RCRI score of 1 (Interquartile range = 2) before their operation with a PMI. Regarding pre‐existing medication use in the year before admission, a substantial proportion of patients were on cardiovascular medications before admission: 55.5% (*n* = 3141) on statins, 53.2% (*n* = 3009) on ACE inhibitors or ARBs, 52.2% (*n* = 2954) on beta‐blockers, and 22.7% (*n* = 1283) on P2Y12 receptor inhibitors. However, only 4.6% (*n* = 261) of patients had a record of filled aspirin prescriptions.

Cardiac complications were observed in 36% (*n* = 2037) of patients during the index admission, while noncardiac complications occurred in 34.5% (*n* = 1952). The most frequently occurring cardiac complications included atrial fibrillation (*n* = 1299 [23%]) and acute heart failure (*n* = 837 [14.8%]), while the most frequently occurring noncardiac complication was respiratory failure (*n* = 1680 [29.7%]) (Table 1).

During the index hospitalization to 3 months post‐discharge, 81.0% (*n* = 4582) of patients were evaluated by a cardiologist, 75.2% (*n* = 4254) by an internal medicine physician, and 57.7% (*n* = 3267) by a family medicine physician. Broken down within this interval of time, during the index hospitalization, 75.6% (*n* = 4277) of patients were evaluated by a cardiologist, 61.4% (*n* = 3477) by an internal medicine physician, and 25.8% (*n* = 1458) by a family medicine physician; and in the 3 months following discharge 55.0% (*n* = 3144) of patients were evaluated by a cardiologist, 57.0% (*n* = 3229) by an internal medicine physician, and 50.6% (*n* = 2862) by a family medicine physician.

### 3.2 Guideline Directed Medical Therapy Following Perioperative Myocardial Infarction

The overall prescription rate of GDMT was relatively low, with 22.1% of patients receiving no GDMT prescriptions (Table 2). Patients filled prescriptions from a mean of 1.88 GDMT drug categories (SD = 1.34), indicating that while most patients were on at least one GDMT drug, they typically received, on average, only one to two classes of GDMT medication. The most common medication classes with filled prescriptions overall were statins (55.8% of patients), ACE inhibitors/ARBs (41.9% of patients), and Beta‐blockers (59.9% of patients). Only 8.14% of patients filled prescriptions for aspirin.

**Table 2:** Characteristics among patients with PMI Who received 0, 1, 2, 3, or 4 GDMT medication classes following PMI.

Characteristics	Number of GDMT Drug Classes with Prescriptions Filled					Adjusted *p*‐value
	0 (*n* = 1251)	1 (*n* = 968)	2 (*n* = 1374)	3 (*n* = 1323)	4 (*n* = 744)	
Age	67.4 (13.2)	68.7 (12.7)	68.7 (12.3)	68.2 (11.4)	66 (10.5)	< 0.001
Length of stay (days)	9 [5–18]	7 [4–13]	8 [4–14]	7 [4–12]	7 [4–12]	< 0.001
Year						< 0.001
2016	313 [25%]	212 [21.9%]	287 [20.9%]	345 [26.1%]	166 [22.3%]	
2017	249 [19.9%]	181 [18.7%]	209 [15.2%]	230 [17.4%]	140 [18.8%]	
2018	161 [12.9%]	99 [10.2%]	184 [13.4%]	175 [13.2%]	127 [17.1%]	
2019	169 [13.5%]	147 [15.2%]	214 [15.6%]	178 [13.5%]	104 [14%]	
2020	191 [15.3%]	190 [19.6%]	272 [19.8%]	221 [16.7%]	118 [15.9%]	
2021	168 [13.4%]	139 [14.4%]	208 [15.1%]	174 [13.2%]	89 [12%]	
Sex = Male	623 [49.8%]	498 [51.4%]	756 [55%]	849 [64.2%]	497 [66.8%]	< 0.001
Urgency of Procedure = Emergent	256 [20.5%]	193 [19.9%]	281 [20.5%]	216 [16.3%]	107 [14.4%]	< 0.001
Type of surgery						< 0.001
Vascular	226 [18.1%]	173 [17.9%]	356 [25.9%]	423 [32%]	294 [39.5%]	
Central Nervous System	42 [3.36%]	32 [3.31%]	31 [2.26%]	35 [2.65%]	14 [1.88%]	
Female Reproductive System	20 [1.6%]	12 [1.24%]	19 [1.38%]	12 [0.907%]	6 [0.806%]	
Gastrointestinal	185 [14.8%]	145 [15%]	179 [13%]	154 [11.6%]	88 [11.8%]	
General Region	21 [1.68%]	32 [3.31%]	39 [2.84%]	28 [2.12%]	18 [2.42%]	
Hepatobiliary and Pancreas	80 [6.39%]	78 [8.06%]	100 [7.28%]	62 [4.69%]	22 [2.96%]	
Lymphatic and Hemic System	19 [1.52%]	11 [1.14%]	15 [1.09%]	17 [1.28%]	5 [0.672%]	
Male Reproductive	12 [0.959%]	14 [1.45%]	8 [0.582%]	17 [1.28%]	9 [1.21%]	
Musculoskeletal, Subcutaneous, and Fascia	438 [35%]	343 [35.4%]	460 [33.5%]	435 [32.9%]	231 [31%]	
Respiratory System	150 [12%]	68 [7.02%]	85 [6.19%]	53 [4.01%]	23 [3.09%]	
Urinary System	31 [2.48%]	37 [3.82%]	55 [4%]	57 [4.31%]	16 [2.15%]	
Other Procedures	27 [2.16%]	23 [2.38%]	27 [1.97%]	30 [2.27%]	18 [2.42%]	
GDMT class prescription filled in 12 months before admission						
Aspirin	36 [2.88%]	37 [3.82%]	46 [3.35%]	75 [5.67%]	67 [9.01%]	< 0.001
P2Y12 receptor inhibitor	135 [10.8%]	114 [11.8%]	245 [17.8%]	433 [32.7%]	356 [47.8%]	< 0.001
ACE inhibitor/ARB	343 [27.4%]	429 [44.3%]	748 [54.4%]	908 [68.6%]	581 [78.1%]	< 0.001
Beta Blocker	337 [26.9%]	433 [44.7%]	785 [57.1%]	897 [67.8%]	502 [67.5%]	< 0.001
Statin	327 [26.1%]	424 [43.8%]	856 [62.3%]	977 [73.8%]	557 [74.9%]	< 0.001
GDMT class prescription filled in 3 months after discharge						
Aspirin	0 [0%]	29 [3%]	84 [6.11%]	156 [11.8%]	192 [25.8%]	< 0.001
P2Y12 receptor inhibitor	0 [0%]	38 [3.93%]	197 [14.3%]	580 [43.8%]	669 [89.9%]	< 0.001
ACE inhibitor/ARB	0 [0%]	208 [21.5%]	570 [41.5%]	851 [64.3%]	744 [100%]	< 0.001
Beta Blocker	0 [0%]	425 [43.9%]	987 [71.8%]	1233 [93.2%]	744 [100%]	< 0.001
Statin	0 [0%]	271 [28%]	929 [67.6%]	1212 [91.6%]	744 [100%]	< 0.001
Evaluation by physician during admission with PMI or in 3 months post discharge						
Cardiologist	971 [77.6%]	778 [80.4%]	1108 [80.6%]	1095 [82.8%]	630 [84.7%]	< 0.001
Family Medicine	675 [54%]	575 [59.4%]	817 [59.5%]	761 [57.5%]	439 [59%]	0.033
Internal Medicine (not elsewhere classified)	914 [73.1%]	731 [75.5%]	1059 [77.1%]	995 [75.2%]	555 [74.6%]	0.21

*Note: p*‐values are computed by an ANOVA test for numeric variables and a chi‐square test for categorical variables and adjusted to a false discovery rate of 0.05 using the Benjamini‐Hochberg procedure. Mean (standard deviation) is reported for each numeric variable, and count [percent of total] is reported for each categorical variable. A Kruskal‐Wallis test was performed, and the median [IQR] was reported for the length of stay due to the non‐normality of this variable. An adjusted *p*‐value less than 0.05 was considered significant.

Abbreviations: GDMT, guideline‐directed medical therapy; PMI, perioperative myocardial infarction.

Cardiology evaluation was significantly associated with an increase in the likelihood of receiving at least one GDMT prescription (78.8% of patients with cardiology evaluation had at least one GDMT prescription vs 74.0% of patients without cardiology evaluation, *p* < 0.001). Patients who received cardiology evaluation were also significantly more likely to receive prescriptions for two or more GDMT medication classes compared to those without cardiology evaluation (61.8% vs. 56.4%, absolute risk difference 5.4%, 95% CI 2.2–8.7%, *p* < 0.001). The Poisson regression model showed that cardiology evaluation was associated with an increased number of GDMT prescriptions (OR = 1.10 [95% CI 1.05–1.17], *p* < 0.001) (Table S6). This indicates that patients who saw a cardiologist filled prescriptions in 1.1 times as many GDMT classes compared to those who did not (mean 1.92 GDMT classes vs 1.73).

### 3.3 Left Heart Catheterization or Cardiac Stress Testing Following Perioperative Myocardial Infarction

Overall, 35.3% of patients underwent left heart catheterization or cardiac stress testing during or within 3 months after the index hospitalization (Table 3). Patients who had a cardiology evaluation were significantly more likely to undergo these procedures compared to those who did not (38.2% vs 23.0%, *p* < 0.001).

**Table 3:** Characteristics among patients with PMI who did or did not receive ischemic evaluation following PMI.

Characteristics	Ischemic Evaluation during hospitalization or in 3 months after discharge		Adjusted *p*‐value
	Yes (*n* = 2000)	No (*n* = 3660)	
Age	66.5 (10.9)	68.8 (12.8)	< 0.001
Length of stay (days)	8 [4–14]	7 [4–14]	0.018
Year			< 0.001
2016	503 [25.2%]	820 [22.4%]	
2017	413 [20.6%]	596 [16.3%]	
2018	265 [13.2%]	481 [13.1%]	
2019	280 [14%]	532 [14.5%]	
2020	307 [15.4%]	685 [18.7%]	
2021	232 [11.6%]	546 [14.9%]	
Sex = Male	1190 [59.5%]	2033 [55.5%]	0.0076
Urgency of Procedure = Emergent	357 [17.8%]	696 [19%]	0.33
Type of surgery			< 0.001
Vascular	657 [32.8%]	815 [22.3%]	
Central Nervous System	35 [1.75%]	119 [3.25%]	
Female Reproductive System	27 [1.35%]	42 [1.15%]	
Gastrointestinal	262 [13.1%]	489 [13.4%]	
General Region	46 [2.3%]	92 [2.51%]	
Hepatobiliary and Pancreas	115 [5.75%]	227 [6.2%]	
Lymphatic and Hemic System	19 [0.95%]	48 [1.31%]	
Male Reproductive	21 [1.05%]	39 [1.07%]	
Musculoskeletal, Subcutaneous, and Fascia	587 [29.4%]	1320 [36.1%]	
Respiratory System	116 [5.8%]	263 [7.19%]	
Urinary System	63 [3.15%]	133 [3.63%]	
Other Procedures	52 [2.6%]	73 [1.99%]	
Evaluation by physician during admission with PMI or in 3 months post discharge			
Cardiologist	1752 [87.6%]	2830 [77.3%]	< 0.001
Family Medicine	1216 [60.8%]	2051 [56%]	0.0012
Internal Medicine (not elsewhere classified)	1541 [77%]	2713 [74.1%]	0.022
Complications during admission with PMI			
Cardiac complication	830 [41.5%]	1207 [33%]	< 0.001
Noncardiac complication	713 [35.6%]	1239 [33.9%]	0.21
Prior cardiac evaluation			
Cardiac stress testing in year before admission with PMI	344 [17.2%]	668 [18.3%]	0.36
Left heart catheterization in year before admission with PMI	178 [8.9%]	527 [14.4%]	< 0.001
Echocardiography in year before admission with PMI	729 [36.4%]	1576 [43.1%]	< 0.001
Echocardiography during admission with PMI	1563 [78.1%]	2037 [55.7%]	< 0.001

*Note: p*‐values are computed by a student's *t*‐test for numeric variables and a chi‐square test for categorical variables and adjusted to a false discovery rate of 0.05 using the Benjamini‐Hochberg procedure. Mean (standard deviation) is reported for each numeric variable, and count [percent of total] is reported for each categorical variable. A Mann‐Whitney *U* test was performed, and the median [IQR] was reported for the length of stay due to the non‐normality of this variable. An adjusted *p*‐value less than 0.05 was considered significant.

Abbreviation: PMI, perioperative myocardial infarction.

The logistic regression model showed that cardiology evaluation was strongly associated with an increased likelihood of catheterization or stress testing (OR = 1.82 [95% CI 1.53–2.18], *p* < 0.001) (Table S7). Other factors significantly associated with the increased likelihood of these procedures included echocardiography during admission. Notably, prior left heart catheterization in the year before the index admission was associated with a decreased likelihood of follow‐up catheterization or stress testing (OR = 0.55 [95% CI 0.45–0.68], *p* < 0.001). While the presence of one or more cardiac complications during admission was positively associated with follow‐up catheterization or stress testing (OR = 1.41 [95% CI 1.24–1.60], *p* < 0.001), the presence of a noncardiac complication was negatively associated with the same outcome (OR = 0.81 [95% CI 0.70–0.93], *p* = 0.002). Evaluation by a family medicine physician was also significantly associated with an increased likelihood of ischemic testing, though with a smaller effect size (OR = 1.22 [95% CI 1.09–1.38], *p* < 0.001).

### 3.4 Echocardiography Following Perioperative Myocardial Infarction

Echocardiography was performed in 71.7% of patients during the index admission or within 3 months of discharge (Table 4). Patients who had cardiology evaluation were significantly more likely to undergo echocardiography compared to those who did not (75.9% vs 53.6%, *p* < 0.001).

**Table 4:** Characteristics among patients with PMI who did or did not receive echocardiography following PMI.

Characteristics	Echocardiography during hospitalization or in 3 months after discharge		Adjusted *p*‐value
	Yes (*n* = 4057)	No (*n* = 1603)	
Age	68.3 (12.1)	67.2 (12.3)	0.0026
Length of stay (days)	9 [5–16]	5 [3–8]	< 0.001
Year			0.79
2016	939 [23.1%]	384 [24%]	
2017	733 [18.1%]	276 [17.2%]	
2018	528 [13%]	218 [13.6%]	
2019	593 [14.6%]	219 [13.7%]	
2020	701 [17.3%]	291 [18.2%]	
2021	563 [13.9%]	215 [13.4%]	
Sex = Male	2290 [56.4%]	933 [58.2%]	0.27
Urgency of Procedure = Emergent	790 [19.5%]	263 [16.4%]	0.011
Type of surgery			< 0.001
Vascular	1155 [28.5%]	317 [19.8%]	
Central Nervous System	119 [2.93%]	35 [2.18%]	
Female Reproductive System	44 [1.08%]	25 [1.56%]	
Gastrointestinal	542 [13.4%]	209 [13%]	
General Region	93 [2.29%]	45 [2.81%]	
Hepatobiliary and Pancreas	222 [5.47%]	120 [7.49%]	
Lymphatic and Hemic System	47 [1.16%]	20 [1.25%]	
Male Reproductive	39 [0.961%]	21 [1.31%]	
Musculoskeletal, Subcutaneous, and Fascia	1270 [31.3%]	637 [39.7%]	
Respiratory System	304 [7.49%]	75 [4.68%]	
Urinary System	132 [3.25%]	64 [3.99%]	
Other Procedures	90 [2.22%]	35 [2.18%]	
Evaluation by physician during admission with PMI or in 3 months post discharge			
Cardiologist	3479 [85.8%]	1103 [68.8%]	< 0.001
Family Medicine	2473 [61%]	794 [49.5%]	< 0.001
Internal Medicine (not elsewhere classified)	3176 [78.3%]	1078 [67.2%]	< 0.001
Complications during admission with PMI			
Cardiac complication	1706 [42.1%]	331 [20.6%]	< 0.001
Noncardiac complication	1693 [41.7%]	259 [16.2%]	< 0.001
Prior cardiac evaluation			
Cardiac stress testing in year before admission with PMI	643 [15.8%]	369 [23%]	< 0.001
Cardiac stress testing during admission with PMI	296 [7.3%]	50 [3.12%]	< 0.001
Left heart catheterization in year before admission with PMI	460 [11.3%]	245 [15.3%]	< 0.001
Left heart catheterization during admission with PMI	1096 [27%]	166 [10.4%]	< 0.001
Echocardiography in year before admission with PMI	1553 [38.3%]	752 [46.9%]	< 0.001

*Note: p*‐values are computed by a student's *t*‐test for numeric variables and a chi‐square test for categorical variables and adjusted to a false discovery rate of 0.05 using the Benjamini‐Hochberg procedure. Mean (standard deviation) is reported for each numeric variable, and count [percent of total] is reported for each categorical variable. A Mann‐Whitney *U* test was performed, and the median [IQR] was reported for the length of stay due to the non‐normality of this variable. An adjusted *p*‐value less than 0.05 was considered significant.

Abbreviation: PMI, perioperative myocardial infarction.

The logistic regression model demonstrated that cardiology evaluation was strongly associated with an increased likelihood of echocardiography (OR = 2.29 [95% CI 1.93–2.71], *p* < 0.001) (Table S8). Other factors significantly associated with increased likelihood of echocardiography included cardiac complications and noncardiac complications. Evaluation by a family medicine physician was also significantly associated with an increased likelihood of echocardiography, though with a smaller effect size (OR = 1.35 [95% CI 1.18–1.54], *p* < 0.001).

## 4 Discussion

### 4.1 Frequency of Cardiology Evaluation Following PMI and Association With Increased Management Frequency

In our retrospective cohort study of management of patients with PMI, nearly one in five patients (19%) with PMI did not receive evaluation by a cardiologist during the index hospitalization or within 3 months after discharge. We observed that cardiology evaluation was associated with increased use of guideline‐recommended management including GDMT, ischemic testing, and echocardiography following PMI.

Patients who saw a cardiologist filled prescriptions in more GDMT classes of medications and were significantly more likely to undergo ischemic testing and echocardiography than those who did not. Furthermore, even in cases where the presence of either internal medicine (not elsewhere specified) or family medicine evaluation was also significantly positively associated with the frequency of these management outcomes, cardiology evaluation demonstrated a larger effect size compared to these other specialties. These findings suggest that the lack of specifically cardiology involvement in PMI management may contribute to decreased frequency of guideline‐recommended care, though we cannot directly determine causation given the retrospective nature of the study.

Our findings regarding the impact of cardiology evaluation on GDMT prescriptions and cardiac testing are consistent with previous studies in patients with nonsurgical myocardial infarction or injury. McCarthy et al. (2020) found that cardiology consultation during hospitalization for a type 2 myocardial infarction is associated with an increased likelihood of coronary angiography, stress testing, and transthoracic echocardiography as well as an increased likelihood of being discharged on a statin, clopidogrel, or a beta blocker [22]. Our study extends these findings to the specific context of perioperative myocardial infarction, where management may be complicated by surgical factors.

### 4.2 Temporal Trends in GDMT Prescription Patterns

Interestingly, we observed a declining trend in the proportion of patients receiving GDMT medications over the study period (2016–2021). This unexpected finding may reflect several possibilities: (1) increasing recognition of Type 2 MI, which may be managed differently than Type 1 MI; (2) growing concerns about bleeding risks associated with antiplatelet therapy in surgical patients; (3) changes in prescribing patterns due to evolving evidence regarding the benefits of GDMT in specific perioperative contexts; or (4) shifts in documentation or billing practices affecting our ability to capture medication use. This trend warrants further investigation in future studies specifically designed to examine temporal changes in PMI management.

### 4.3 Factors Associated With Likelihood of Ischemic Evaluation Following PMI

The ischemic evaluation model revealed notable patterns in the frequency of left heart catheterization or cardiac stress testing after PMI. Diagnoses that suggested a noncardiac etiology (e.g., sepsis, hypovolemic shock, respiratory failure, or pulmonary embolism) that occurred during the index admission with PMI were associated with a lower likelihood of catheterization or cardiac stress testing. In contrast, cardiac complications, such as heart failure and cardiogenic shock, were positively associated with ischemic evaluation. This pattern, assuming the complications occurred before the PMI, suggests that an increased likelihood of presumed Type 1 MI (i.e., due to plaque rupture and coronary thrombosis) drives further cardiac workup of PMI, while PMI likely due to other causes may not lead to additional coronary evaluation [23]. Furthermore, the presence of left heart catheterization in the year before the index admission was negatively associated with repeat left heart catheterization or cardiac stress testing following PMI, suggesting that knowledge of information regarding coronary disease from prior testing reduces repeat evaluation.

### 4.4 Echocardiography Is Frequent Following PMI

The echocardiography model showed predominantly positive coefficients across various cardiac and noncardiac comorbidities and complications. This suggests that providers generally view echocardiography as a valuable tool to assess myocardial function in the context of PMI, regardless of the presumed etiology, and this is supported by management guidelines [24]. In addition, the widespread use of echocardiography, with 71.7% of patients undergoing the procedure, may be driven by the need for comprehensive cardiac assessment in these high‐risk patients and the relatively greater access to echocardiography compared to left heart catheterization or cardiac stress testing. This widespread use of echocardiography is also consistent with 2019 Appropriate Use Criteria [24].

### 4.5 Heterogeneity in Management and Potential Implications

Our study reveals significant heterogeneity in the management of PMI, highlighting the current lack of consensus on optimal care. The observed differences between patients who did and did not receive cardiology evaluation reflect potential disparities in treatment approaches and underscore the critical need for further research to determine best practices and improve outcomes. Patients who experience PMI are at increased risk of subsequent cardiac events [6], emphasizing the importance of effective management strategies for this high‐risk population.

Our findings regarding the potential impact of cardiology evaluation can be considered alongside previous literature examining the implementation of cardiology recommendations. Marques et al. studied 589 hospitalized patients who received cardiology consultations and found that when the primary teams did not adhere to cardiology recommendations, patients had significantly worse outcomes (OR = 10.25 [95% CI 4.45–23.62]) [25]. While their study focused on adherence to recommendations among patients who received cardiology consultation rather than comparing patients with and without cardiology evaluation as in our study, their findings support the broader concept that optimal consultation and implementation of cardiology expertise is associated with better care. Their identification of specific factors associated with increased adherence to recommendations, such as follow‐up notes and verbal reinforcement, suggests potential strategies to improve the implementation of cardiology recommendations in the post‐PMI setting.

It is also worth noting that the evidence base for PMI management remains limited. To date, the only medication specifically evaluated for postoperative MI is dabigatran, as investigated in the MANAGE trial [26]. Our findings provide a foundation for future studies to explore the effectiveness of different management strategies and develop evidence‐based guidelines for PMI management.

### 4.6 Limitations

Our study has several limitations. The use of claims data limits our ability to assess the clinical decision‐making process and the appropriateness of interventions in individual cases. Therefore, it is difficult to comment on the appropriateness of utilization or lack of utilization of certain management strategies following PMI in this study. Additionally, the lack of additional clinical confirmation of myocardial infarctions and strokes/transient ischemic attacks may have led to misclassification of these events. However, these codes are moderately robust at capturing clinically validated events, as the ICD codes have been validated in prior studies (Table S9) [27, 28, 29, 30, 31, 32, 33]. The capture of cardiac testing may also be incomplete due to our reliance on procedural terminology codes. However, while extensive validation studies of such codes are limited, a recent study examining intraoperative transesophageal echocardiography (TEE) Current Procedural Terminology codes found them to be 99.88% sensitive and 100% specific for the identification of TEE [34]. This suggests procedural codes adequately capture the implementation of major cardiac diagnostic tests.

Our medication analysis likely underestimates the true use of certain therapies, particularly aspirin, as we cannot capture over‐the‐counter medication purchases through claims data. Similarly, we could not assess for medication contraindications, which may have influenced GDMT prescription rates. We are unable to make conclusions about causation, given the retrospective nature of our study. As we excluded patients without insurance coverage in the 3 months following discharge from their hospitalization, we, by definition, excluded patients who died during this period.

## 5 Conclusions

In conclusion, our study demonstrates variations in the frequency of guideline‐based management strategies for PMI and their association with specialty consultation. By highlighting the positive impact of cardiology evaluation and identifying gaps in current practice, these findings may inform efforts to enhance the quality of care for this high‐risk patient population.

Future research should focus on evaluating the impact of these management strategies on long‐term outcomes for PMI patients. In addition, investigating barriers to implementing guideline‐recommended care and developing interventions to improve adherence to GDMT and appropriate use of diagnostic procedures could help optimize care for patients following PMI.

## Ethics Statement

Ethical approval was not sought for the present study because the research utilized secondary data that does not contain individually identifiable information.

## Conflicts of Interest

The authors declare no conflicts of interest.

## Supporting information

CLC70120

CLC70120

## Data Availability

The data that support the findings of this study are available from IBM MarketScan Research Databases. Restrictions apply to the availability of these data, which were used under license for this study. Data are available from https://www.ibm.com/watson/health/resources/ipv-opv/ with the permission of IBM MarketScan Research Databases. Data used in this study were obtained from IBM MarketScan Research Databases under a licensed agreement. While these data support the study's findings, they are not publicly accessible. Researchers interested in accessing these data must contact IBM MarketScan directly and are subject to licensing restrictions.
