# Supplementary material for: Frequency and Impact of Cardiology Evaluation Following Perioperative Myocardial Infarction
Source: Clin Cardiol. 2025 Mar 21;48(3):e70120. doi: 10.1002/clc.70120 (PMC11926558; doi:10.1002/clc.70120)
Supplement: Supplementary file 2 — CLC70120 [file CLC-48-e70120-s001.docx]

**SUPPLEMENTAL MATERIAL**

Table S1. ICD-10 procedure codes for major operating room procedures

Please see “AdditionalSupplementalTables.xlsx”.

Table S2. National Drug Code numbers for medications

Please see “AdditionalSupplementalTables.xlsx”.

Table S3. Current Procedural Terminology codes for cardiac evaluation procedures

| **Procedure Type** | **Current Procedural Terminology codes** |
| --- | --- |
| Stress Testing | 93015, 93016, 93017, 93018, 93350, 93351, C8928, C8930, G8961, G8962, G8963, G8964, G8965, G8966, 78451, 78452, 78453, 78454, 78460, 78461, 78464, 78465, 78472, 78473, 78481, 78483, 78491, 78492, 75559, 75560, 75563, 75564, C9763, 78430, 78431, 78434, C7519, C7522, C7524, C7526, C7552, C7553, C7528, C7529 |
| Left Heart Catheterization | 93452, 93453, 93454, 93455, 93456, 93457, 93458, 93459, 93460, 93461, 93462 |
| Echocardiography | 93303, 93304, 93306, 93307, 93308, 93312, 93313, 93314, 93315, 93316, 93317, 93318, 93319, 93320, 93321, 93325, 93350, 93351, 93352, 93355, 93356 |

Table S4. ICD-9 and ICD-10 codes for diagnoses

| **Prior Comorbidities** | |
| --- | --- |
| TIA or Stroke: | 430, 431, 433.x1, 434 (excluding 434.x0), 435, 436, G46.3, G46.4, G46.5, G46.6, G46.7, 163.00, 163.011, 163.012, 163.013, 163.019, 163.02, 163.031, 163.032, 163.033, 163.039, 163.09, 163.10, 163.111, 163.112, 163.113, 163.119, 163.12, 163.131, 163.132, 163.133, 163.139, 163.19, 163.20, 163.211, 163.212, 163.213, 163.219, 163.22, 163.231, 163.232, 163.233, 163.239, 163.29, 163.30, 163.311, 163.312, 163.313, 163.319, 163.321, 163.322, 163.323, 163.329, 163.331, 163.332, 163.333, 163.339, 163.341, 163.342, 163.343, 163.349, 163.39, 163.40, 163.411, 163.412, 163.413, 163.419, 163.421, 163.422, 163.423, 163.429, 163.431, 163.432, 163.433, 163.439, 163.441, 163.442, 163.443, 163.449, 163.49, 163.50, 163.511, 163.512, 163.513, 163.519, 163.521, 163.522, 163.523, 163.529, 163.531, 163.532, 163.533, 163.539, 163.541, 163.542, 163.543, 163.549, 163.59, 163.6, 163.81, 163.89, 163.9 160.00, 160.01, 160.02, 160.10, 160.11, 160.12, 160.2, 160.30, 160.31, 160.32, 160.4, 160.50, 160.51, 160.52, 160.6, 160.7, 160.8, 160.9, 161.0, 161.1, 161.2, 161.3, 161.4, 161.5, 161.6, 161.8, 161.9, G45.8, G45.9, H34.0 |
| Chronic Kidney Disease stage 3 or higher: | 403.01, 403.11, 403.91, 404.02, 404.03, 404.12, 404.13, 404.92, 404.92, 404.93, 585.3, 585.4, 585.5, 585.6, 586, V420, V451, V56, I120, I1311, I132, N183, N184, N185, N186, N19, Z49, Z940, Z992 |
| Heart failure: | 398.91, 402.01, 402.11, 402.91, 404.01, 404.03, 404.11, 404.13, 404.91, 404.93, 425.4, 425.5, 425.6, 425.7, 425.8, 425.9, 428, I099, I110, I130, I132, I255, I420, 1425, 1426, 1427, 1428, I429, I43, I50, P290 |
| Ischemic Heart Disease: | 410, 412, 413, 414, I20, I21, I22, I25 |
| **Cardiac complications occurring during index hospitalization** | |
| Supraventricular Tachycardia: | 427.0, I471 |
| Cardiogenic Shock: | 785.51, R570, T811XA |
| Atrial Fibrillation: | 427.31, I480, I481, I482, I4891 |
| acute heart failure: | 428.21, 428.23, 415.0, 428.31, 428.33, 428.41, 428.43, I9713, I5033, I5031, I5023, I5021, I5041, I50811, I50813, I5043 |
| **Noncardiac complications occurring during index hospitalization** | |
| Severe Sepsis (without septic shock): | 995.92, R6520, A40 |
| Septic Shock: | 785.52, R6521, T8112XA |
| Hypovolemic Shock: | 785.59, R571 |
| Respiratory failure: | 799.1, 518.81, 518.83, 518.84, 518.51, 518.53, J96, J9582, R092 |
| Pulmonary Embolism: | 415.1, I26 |
| **Types of myocardial infarction diagnoses** | |
| STEMI | 410.0, 410.1, 410.2, 410.3, 410.4, 410.5, 410.6, 410.8, 410.9, I210, I211, I212, I213 |
| NSTEMI | 410.71, I214 |
| Type 2 MI | I21A1 |
|  |  |

Table S5. Clinical Classifications Software Refined Categories for RCRI high risk surgeries

| **Category of surgical procedure** | **Clinical Classifications Software Refined Category codes** |
| --- | --- |
| Intrathoracic surgery | RES008, RES009 |
| Intraabdominal surgery | GIS009, GIS010, GIS011, GIS012, GIS013, GIS014, GIS015 |
| Suprainguinal vascular surgery | CAR014 |

Table S6: Model coefficients for Poisson regression model of guideline directed medical therapy following PMI

|  |  |  |
| --- | --- | --- |
| **Covariate** | **Adjusted OR [95% CI]** | **Adjusted p-Value** |
| **Age** | 1.00 [0.99-1.00] | <0.001 |
| **Female** | 0.92 [0.89-0.96] | <0.001 |
| **Length of Stay** | 1.00 [0.99-1.00] | <0.001 |
| **Emergent Procedure** | 0.93 [0.88-0.98] | 0.007 |
| **Vascular Procedure** | 1.56 [1.36-1.78] | <0.001 |
| **Central Nervous System Procedure** | 1.29 [1.08-1.54] | 0.006 |
| **Female Reproductive System Procedure** | 1.24 [0.99-1.54] | 0.070 |
| **Gastrointestinal System Procedure** | 1.31 [1.14-1.50] | <0.001 |
| **General Region Procedure** | 1.44 [1.21-1.72] | <0.001 |
| **Hepatobiliary and Pancreas Procedure** | 1.25 [1.07-1.45] | 0.006 |
| **Lymphatic and Hemic System Procedure** | 1.29 [1.03-1.61] | 0.033 |
| **Male Reproductive System Procedure** | 1.30 [1.04-1.62] | 0.029 |
| **Musculoskeletal, Subcutaneous Tissue, and Fascia Procedure** | 1.33 [1.16-1.53] | <0.001 |
| **Respiratory System Procedure** | 1.07 [0.92-1.26] | 0.42 |
| **Urinary System Procedure** | 1.36 [1.16-1.60] | <0.001 |
| **Other Procedure** | 1.44 [1.21-1.73] | <0.001 |
| **Cardiology Evaluation** | 1.10 [1.05-1.17] | <0.001 |
| **Family Medicine Evaluation** | 1.06 [1.02-1.10] | 0.004 |
| **Internal Medicine (Not elsewhere classified) Evaluation** | 1.01 [0.96-1.06] | 0.73 |
| **ACEi/ARB Prescription Filled in Year Prior to Admission with PMI** | 1.39 [1.33-1.45] | <0.001 |
| **Beta blocker Prescription Filled in Year Prior to Admission with PMI** | 1.19 [1.14-1.24] | <0.001 |
| **Aspirin Prescription Filled in Year Prior to Admission with PMI** | 0.99 [0.91-1.07] | 0.74 |
| **Statin Prescription Filled in Year Prior to Admission with PMI** | 1.36 [1.30-1.43] | <0.001 |
| **P2Y12 receptor inhibitor Prescription Filled in Year Prior to Admission with PMI** | 1.17 [1.12-1.22] | <0.001 |

Note: A Benjamini-Hochberg adjusted P-value less than 0.05 was considered significant.

Abbreviations: OR, odds ratio; CI, confidence interval; PMI, perioperative myocardial infarction

Table S7: Model coefficients for logistic regression model of ischemic evaluation following PMI

|  |  |  |
| --- | --- | --- |
| **Covariate** | **Adjusted OR [95% CI]** | **Adjusted p-Value** |
| **Age** | 0.98 [0.98-0.99] | <0.001 |
| **Female** | 0.86 [0.76-0.97] | 0.033 |
| **Length of Stay** | 0.99 [0.99-1.00] | 0.006 |
| **Emergent Procedure** | 0.82 [0.70-0.95] | 0.025 |
| **Vascular Procedure** | 1.01 [0.67-1.50] | 0.98 |
| **Central Nervous System Procedure** | 0.35 [0.20-0.60] | <0.001 |
| **Female Reproductive System Procedure** | 0.81 [0.43-1.51] | 0.54 |
| **Gastrointestinal System Procedure** | 0.70 [0.46-1.06] | 0.14 |
| **General Region Procedure** | 0.72 [0.42-1.23] | 0.28 |
| **Hepatobiliary and Pancreas Procedure** | 0.67 [0.43-1.05] | 0.12 |
| **Lymphatic and Hemic System Procedure** | 0.51 [0.26-1.00] | 0.086 |
| **Male Reproductive System Procedure** | 0.73 [0.37-1.44] | 0.40 |
| **Musculoskeletal, Subcutaneous Tissue, and Fascia Procedure** | 0.63 [0.42-0.94] | 0.051 |
| **Respiratory System Procedure** | 0.57 [0.36-0.89] | 0.033 |
| **Urinary System Procedure** | 0.63 [0.38-1.05] | 0.118 |
| **Other Procedure** | 0.96 [0.57-1.64] | 0.92 |
| **Cardiac Complication During Admission with PMI** | 1.41 [1.24-1.60] | <0.001 |
| **Noncardiac Complication During Admission with PMI** | 0.81 [0.70-0.93] | 0.002 |
| **Cardiology Evaluation** | 1.82 [1.53-2.18] | <0.001 |
| **Family Medicine Evaluation** | 1.22 [1.09-1.38] | <0.001 |
| **Internal Medicine (Not elsewhere classified) Evaluation** | 0.91 [0.79-1.06] | 0.22s |
| **Cardiac Stress Test in Year Prior to Admission with PMI** | 1.14 [0.96-1.34] | 0.137 |
| **Left Heart Catheterization in Year Prior to Admission with PMI** | 0.55 [0.45-0.68] | <0.001 |
| **Echocardiography in Year Prior to Admission with PMI** | 0.86 [0.75-0.99] | 0.037 |
| **Echocardiography During Admission with PMI** | 2.74 [2.39-3.14] | <0.001 |
| **Prior Ischemic Heart Disease** | 1.08 [0.95-1.24] | 0.256 |
| **Prior Heart Failure** | 0.82 [0.70-0.96] | 0.033 |
| **Prior Insulin-Dependent Diabetes** | 1.21 [1.03-1.42] | 0.038 |
| **Prior Stroke or Transient Ischemic Attack** | 1.19 [0.96-1.47] | 0.16 |
| **Prior Chronic Kidney Disease stage 3 or higher** | 0.86 [0.74-1.01] | 0.12 |

Note: A Benjamini-Hochberg adjusted P-value less than 0.05 was considered significant.

Abbreviations: OR, odds ratio; CI, confidence interval

Table S8: Model coefficients for logistic regression model of echocardiography following PMI

|  |  |  |
| --- | --- | --- |
| **Covariate** | **Adjusted OR [95% CI]** | **Adjusted p-Value** |
| **Age** | 1.01 [1.01-1.02] | <0.001 |
| **Female** | 1.07 [0.94-1.23] | 0.36 |
| **Length of Stay** | 1.04 [1.03-1.05] | <0.001 |
| **Emergent Procedure** | 1.04 [0.87-1.23] | 0.70 |
| **Vascular Procedure** | 0.28 [0.18-0.44] | <0.001 |
| **Central Nervous System Procedure** | 0.28 [0.16-0.50] | <0.001 |
| **Female Reproductive System Procedure** | 0.16 [0.08-0.32] | <0.001 |
| **Gastrointestinal System Procedure** | 0.19 [0.12-0.30] | <0.001 |
| **General Region Procedure** | 0.16 [0.09-0.29] | <0.001 |
| **Hepatobiliary and Pancreas Procedure** | 0.17 [0.10-0.27] | <0.001 |
| **Lymphatic and Hemic System Procedure** | 0.22 [0.11-0.44] | <0.001 |
| **Male Reproductive System Procedure** | 0.17 [0.08-0.34] | <0.001 |
| **Musculoskeletal, Subcutaneous Tissue, and Fascia Procedure** | 0.16 [0.10-0.25] | <0.001 |
| **Respiratory System Procedure** | 0.16 [0.10-0.27] | <0.001 |
| **Urinary System Procedure** | 0.20 [0.12-0.34] | <0.001 |
| **Other Procedure** | 0.21 [0.12-0.39] | <0.001 |
| **Cardiac Complication During Admission with PMI** | 1.91 [1.64-2.23] | <0.001 |
| **Noncardiac Complication During Admission with PMI** | 2.42 [2.04-2.87] | <0.001 |
| **Cardiology Evaluation** | 2.29 [1.93-2.71] | <0.001 |
| **Family Medicine Evaluation** | 1.35 [1.18-1.54] | <0.001 |
| **Internal Medicine (Not elsewhere classified) Evaluation** | 1.10 [0.94-1.29] | 0.26 |
| **Cardiac Stress Test in Year Prior to Admission with PMI** | 0.84 [0.70-1.00] | 0.064 |
| **Cardiac Stress Test During Admission with PMI** | 2.24 [1.62-3.09] | <0.001 |
| **Left Heart Catheterization in Year Prior to Admission with PMI** | 1.05 [0.85-1.29] | 0.70 |
| **Left Heart Catheterization During Admission with PMI** | 2.71 [2.24-3.26] | <0.001 |
| **Echocardiography in Year Prior to Admission with PMI** | 0.65 [0.55-0.76] | <0.001 |
| **Prior Ischemic Heart Disease** | 0.81 [0.69-0.94] | 0.007 |
| **Prior Heart Failure** | 1.19 [1.00-1.41] | 0.070 |
| **Prior Insulin-Dependent Diabetes** | 1.18 [0.98-1.42] | 0.091 |
| **Prior Stroke or Transient Ischemic Attack** | 1.01 [0.79-1.29] | 0.93 |
| **Prior Chronic Kidney Disease Stage 3 or higher** | 1.05 [0.88-1.26] | 0.65 |

Note: A Benjamini-Hochberg adjusted P-value less than 0.05 was considered significant.

Abbreviations: OR, odds ratio; CI, confidence interval; PMI, perioperative myocardial infarction

Table S9. Previously published validation statistics for ICD codes

| **Diagnosis** | **ICD Codes** | **Sensitivity** | **Specificity** | **Positive Predictive Value** | **Negative Predictive Value** |
| --- | --- | --- | --- | --- | --- |
| Myocardial infarction^1,2^ | **ICD-9 Code** 410 or **ICD-10 Codes** I21 and I22 | 61 - 83.99 | N/A | 79 - 96.88 | N/A |
| Myocardial infarction^3–5^ | **ICD-9** **Code** 410 | 80.9 - 94.37 | 92.84 - 99.79 | 54.6 - 88.54 | 93.03 - 99.98 |
| Stroke/TIA^6^ | **ICD-10 Codes** G46.3, G46.4, G46.5, G46.6, G46.7, 163.00, 163.011, 163.012, 163.013, 163.019, 163.02, 163.031, 163.032, 163.033, 163.039, 163.09, 163.10, 163.111, 163.112, 163.113, 163.119, 163.12, 163.131, 163.132, 163.133, 163.139, 163.19, 163.20, 163.211, 163.212, 163.213, 163.219, 163.22, 163.231, 163.232, 163.233, 163.239, 163.29, 163.30, 163.311, 163.312, 163.313, 163.319, 163.321, 163.322, 163.323, 163.329, 163.331, 163.332, 163.333, 163.339, 163.341, 163.342, 163.343, 163.349, 163.39, 163.40, 163.411, 163.412, 163.413, 163.419, 163.421, 163.422, 163.423, 163.429, 163.431, 163.432, 163.433, 163.439, 163.441, 163.442, 163.443, 163.449, 163.49, 163.50, 163.511, 163.512, 163.513, 163.519, 163.521, 163.522, 163.523, 163.529, 163.531, 163.532, 163.533, 163.539, 163.541, 163.542, 163.543, 163.549, 163.59, 163.6, 163.81, 163.89, 163.9 160.00, 160.01, 160.02, 160.10, 160.11, 160.12, 160.2, 160.30, 160.31, 160.32, 160.4, 160.50, 160.51, 160.52, 160.6, 160.7, 160.8, 160.9, 161.0, 161.1, 161.2, 161.3, 161.4, 161.5, 161.6, 161.8, 161.9, G45.8, G45.9, H34.0 | 95.5 | 97.9 | 49.1 | 99.9 |
| Stroke/TIA^7^ | **ICD-9 Codes** 430, 431, 433.x1, 434 (excluding 434.x0), 435, 436 | 82 - 98 | 92 - 95 | 80 - 90 | N/A |

**REFERENCES**

1. Merry AHH, Boer JMA, Schouten LJ, Feskens EJM, Verschuren WMM, Gorgels APM, van den Brandt PA. Validity of coronary heart diseases and heart failure based on hospital discharge and mortality data in the Netherlands using the cardiovascular registry Maastricht cohort study. *Eur. J. Epidemiol.* 2009;24:237–247.

2. Pajunen P, Koukkunen H, Ketonen M, Jerkkola T, Immonen-Räihä P, Kärjä-Koskenkari P, Mähönen M, Niemelä M, Kuulasmaa K, Palomäki P, et al. The validity of the Finnish Hospital Discharge Register and Causes of Death Register data on coronary heart disease. *Eur. J. Cardiovasc. Prev. Rehabil. Off. J. Eur. Soc. Cardiol. Work. Groups Epidemiol. Prev. Card. Rehabil. Exerc. Physiol.* 2005;12:132–137.

3. Kennedy GT, Stern MP, Crawford MH. Miscoding of hospital discharges as acute myocardial infarction: implications for surveillance programs aimed at elucidating trends in coronary artery disease. *Am. J. Cardiol.* 1984;53:1000–1002.

4. Austin PC, Daly PA, Tu JV. A multicenter study of the coding accuracy of hospital discharge administrative data for patients admitted to cardiac care units in Ontario. *Am. Heart J.* 2002;144:290–296.

5. Pladevall M, Goff DC, Nichaman MZ, Chan F, Ramsey D, Ortíz C, Labarthe DR. An assessment of the validity of ICD Code 410 to identify hospital admissions for myocardial infarction: The Corpus Christi Heart Project. *Int. J. Epidemiol.* 1996;25:948–952.

6. Columbo JA, Daya N, Colantonio LD, Wang Z, Foti K, Hyacinth HI, Johansen MC, Gottesman R, Goodney PP, Howard VJ, et al. Derivation and Validation of ICD-10 Codes for Identifying Incident Stroke. *JAMA Neurol.* 2024;81:875–881.

7. Tirschwell DL, Longstreth WT. Validating administrative data in stroke research. *Stroke*. 2002;33:2465–2470.
